# Hyperactivation of IL-6/STAT3 pathway leaded to the poor prognosis of post-TACE HCCs by HIF-1α/SNAI1 axis-induced epithelial to mesenchymal transition

**DOI:** 10.7150/jca.35631

**Published:** 2020-01-01

**Authors:** Xiaohong Gai, Peng Zhou, Meng Xu, Zhikui Liu, Xin Zheng, Qingguang Liu

**Affiliations:** 1 Department of Hepatobiliary Surgery, the First Affiliated Hospital of Xi'an Jiaotong University, Xi'an, Shaanxi 710061, China; 2 Department of General Surgery, Xian NO.3 Hospital, Xi'an, Shaanxi 710001, China

**Keywords:** IL-6/STAT3 signaling, HCC, TACE, HIF-1α, SNAI1

## Abstract

Transarterial chemoembolization (TACE) has been considered the standard treatment for intermediate-stage hepatocellular carcinoma according to BCLC algorithm. However, it has been unclear about the TACE-related predictive bio-markers and underlying molecular mechanisms. This investigation revealed that HCCs with higher HIF-1α suffered from unfavorable OS after TACE. mRNA expression microarray revealed that HIF-1α was potential target of p-STAT3 which was verified by ChIP and immunoblotting assay. Activation of IL-6/STAT3/HIF-1α signaling was found to promote EMT and chemoresistance to Doxorubicin *in vitro* and *in vivo* by regulating SNAI1. Hypoxia did not enhance HIF-1α expression and influence cell growth and chemoresistence to Doxorubicin in HCC cells when STAT3 expression was abolished. Taken together, HIF-1α overexpression in HCC tissues predicted the unfavorable outcome of HCCs after TACE and IL-6/STAT3 pathway resulted in EMT induced-metastases and chemoresistance of HCC after TACE through HIF-1α/SNAI1 axis.

## Introduction

Hepatocellular carcinoma (HCC) is one of the leading malignant tumor with the incidence of approximately 850,000 new cases each year and the second cause of cancer-related deaths all through the world^1^. Due to the absence of surveillance for early detection and typical symptoms at the early stage, HCC was usually found at the advanced stage and closely related with cirrhosis decompensation, which leaded to curative surgical therapies (ablation, liver resection, or liver transplantation) ineligible for most of HCCs. Although a variety of potentially effective therapeutic options were evaluated in HCC patients at the intermediate stage, transarterial chemoembolization (TACE) was the only therapy displaying a notable survival benefit^2^, ^3^ and the largest global observational study about HCC up to date revealed that approximately half of HCC patients underwent TACE at some time point during the course of the disease^4^. Unfortunately, the outcome of TACE for HCCs remained unsatisfied with the median overall survival time of 19.4 months and 3-year survival rate of 40.4%^5^. Although there was limited understanding about predictors of TACE outcome and the mechanism of HCC recurrence and metastases after TACE, hypoxia condition secondary to transarterial embolization (TAE) has been considered to play the critical role^6^, ^7^.

Interleukin 6 (IL-6) is a pleiotropic cytokine which is found to be secreted by various cell types including immune cells, fibroblasts, endothelial cells and tumor cells^8^, ^9^. It has been revealed that IL-6 binds with IL-6R to form the IL-6/IL-6R complex. IL-6/IL-6R complex consequently leads to GP130 dimerization, which activates Janus kinase (JAK) and then phosphorylates signal transducer and activator of transcription 3 (STAT3). p-STAT3 forms homo- or heterodimers, and then is trans-located to the cell nucleus. In nucleus, p-STAT3 is bound with the promoter of its effector genes contributing to stemness maintenance^10^, EMT^11^, autophagy^12^, inflammation^13^, angiogenesis^14^ and immune reaction^15^ in cancer microenvironment. IL-6/STAT3 pathway was also found hyperactivated aberrantly in HCC microenvironment and involved in hepatocarcinogenesis^16^-^18^. Our previous report revealed that p-STAT3 protein activated by IL-6 increased TIMP-1 secreted by HCC cells and then leaded to the trans-differentiation from hepatic stellate cells to cancer-associated fibroblasts (CAFs)^9^.

Persistent hepatic inflammation caused by infection of hepatitis B and hepatitis C played a critical role in the initiation and progression of HCC^19^-^21^. IL-6/STAT3 signal was found to be involved closely in the process of hepatocarcinogenesis driven by hepatitis associated inflammation^22^-^26^. Intriguingly, hypoxia and inflammation caused by embolization have been considered to promote growth and metastases of residual HCC cells^27^-^30^.

Hypoxia inducible factor-1α (HIF-1α) was a member of hypoxia inducible factor system also composed of HIF2α/EPAS1, HIF3α, HIF1β/ARNT1, ARNT2 and ARNT3)^31^. Previous studies revealed that HIF-1α protein was constitutively degraded by ubiquitination and proteasomal degradation by von Hippel-Lindau tumor suppressor protein (pVHL), an E3 ligase, and maintained at the limited basal level on normoxia with a very short half-life (approximately 5min)^32^. However, a growing body of evidences proved that HIF-1α expression was apparently increased in human HCC samples and related with poor prognosis^33^-^36^. And further studies supported the involvement of HIF-1α on the different malignant behaviors of HCC, such as promoting angiogenesis, deregulation cellular energetics, accelerating invasion and metastasis, and resisting cell death. Under hypoxia, because of absence of oxygen, the hydroxylation and proteasomal degradation of HIF-1α were abolished and there was lots of stabilized HIF-1α protein trans-located into nucleus as the transcription factor. Thus, it was assumed that hypoxia secondary to TACE resulted in HIF-1α overexpression. However, due to cancer heterogeneity, HCC patients suffered from quite different outcome after TACE, which indicated that hypoxia was not the only reason for aberrant overexpression of HIF-1α after TACE.

In the present investigation, we demonstrated initially that HCC patients with overexpression of p-STAT3 in tumor tissues suffered from unfavorable survival after TACE. And mechanistic studies revealed that IL-6/STAT3 pathway mediated positively HIF-1α/SNAI1 axis, which induced EMT phenotype of HCC cells. Consequently, EMT was responsible for HCC chemoresistence to Doxorubicin and metastasis after TACE.

## Materials and methods

### Patients and specimens

The investigation was approved by the institutional review board and human ethics committee of the First Hospital of Xian Jiaotong University. Both tumor and adjacent liver tissues were obtained from 86 HCC patients by liver biopsy before TACE from January 2012 and February 2013. All patients had undergone TACE (5-15 ml lipiodol and 10mg/m^2^ Doxorubicin (DOX)) 1-2 months. All HCC patients received routine follow-up and the relevant information was reviewed by two investigators independently, and any disagreement was settled by consensus. Tumor response was evaluated by contrast-enhanced CT scan after TACE. Tumor response was divided into four grades including complete response (CR), partial response (PR), stable disease (SD) and progressive disease (PD), based on the Modified Response Evaluation Criteria In Solid Tumors (mRECIST)^37^. Overall survival (OS) was calculated from the date of TACE to the date of death or last follow-up. The follow-up information was obtained from all patients because of periodic TACE. The detailed informed consent was signed by each patient enrolled in this investigation. Clinicopathologic features for all HCC patients were presented in Table 1.

### Immunohistochemistry (IHC) staining

IHC staining was performed as introduced previously^38^. Briefly speaking, all slides were dewaxed, dehydrated and rehydrated. The primary antibodies against p-STAT3 (Catalog No. : ab32143, Abcam, MA, USA), HIF-1α (Catalog No. : ab51608, Abcam, MA, USA) and SNAI1 (Catalog No. : ab53519, Abcam, MA, USA) were added to the sections and incubating at 4℃ overnight respectively and then the biotinylated secondary antibodies (BOSTER Biology Technology co., Wuhan, China) were used. We designed IHC scoring criteria by staining intensity and percentage of positive staining tumor cells. Staining intensity was divided into four grades: 0, none; 1, weak; 2, moderate; 3, strong, while percentage of positive staining tumor cells was classified into 5 grades: 0 (<5%), 1 (6%-25%), 2 (26%-50%), 3 (51%-75%), and 4 (>75%).

### Cell culture and hypoxia environment

There were 4 kinds of HCC cell lines (Huh7, Hep3B, HepG2 and MHCC97h) and immortalized human hepatocyte LO2 used in this study. All cells were cultured in Dulbecco's Modified Eagle's Medium supplemented with 10% FBS in an incubator with 5% CO2 at 37℃ under standard culture condition. HCC cells were exposed to CoCL2 or hypoxia condition (37℃ humidified incubator with the gas mixture comprised with 1% O2, 5% CO2 and 94% N2 for 5 min).

### RNA extraction and quantitative real-time polymerase chain reaction (qRT-PCR)

Total RNA was extracted from HCC cells using TRIzol reagent (Invitrogen) which was reversely transcribed into cDNA for qRT-PCR. qRT-PCR was conducted using Fast SYBR green Master Mix (Applied Biosystems) to assess the level of target genes with GAPDH as endogenous controls. The primes used here were listed as following: STAT3 Forward: GTGTGACACCGTAAGTGGCT, Reverse: GACATCGGCAGGTCAATGGT; HIF-1α Forward: CTCCATTACCCACCGCTGAA, Reverse: GTAGCTGCATGATCGTCTGG; GAPDH Forward: ACCACAGTCCATGCCATCAC, Reverse: TCCACCACCCTGTTGCTGTA.

### Immunoblot assay

HCC cells were lysed in RIPA buffer with EDTA-free Cocktail and then centrifuged to obtain the supernatant. Protein sample were separated on SDS polyacrylamide gels and then blocked with 3% BSA in TBST. All blots were incubated with primary antibodies respectively at 4 °C overnight and washed twice with TBS. Protein samples were incubated for 2h with HRP-conjugated secondary antibodies, respectively. After washed with TBS twice, all blots were detected by the HyGLO HRP detection kit (Denville, NJ, USA). Protein expression level was normalized to β-actin expression.

### Establishment of STAT3 knockdown and HIF-1α knockdown cells

For STAT3 knockdown assay, Huh7 cells were transfected with the STAT3-specific siRNAs (Catalog No.: sc-156142 from Santa Cruz Biotechnology, USA) using Lipofectamine 2000 (Invitrogen, USA) to be Huh7 HIF-1α siRNA cells, while the scramble siRNA sequences were transfected into Huh7 cells as the Huh7 Scr siRNA cells. Meanwhile, by the same method, SNAI1 siRNA sequences from Santa Cruz Biotechnology (Catalog No.: sc-38398) was used to repress the expression of SNAI1 in Huh7 HiF-1α cells.

### mRNA expression profiling assay for HCC cells

STAT3 siRNA sequences (Catalog No.:sc-29493) were purchased from Santa Cruz Biotechnology (CA, USA). SMMC7721 cells were transfected with STAT3 siRNA to be SMMC7721 STAT3 siRNA cells which was confirmed to express the low level of STAT3 by both qRT-PCR and Western immunoblotting. Meanwhile, SMMC7721 cells was transfected with control siRNA (Catalog No.:sc-37007) as SMMC7721 Scr siRNA cells. The RNA was isolated from both SMMC7721 STAT3 siRNA and SMMC7721 Scr siRNA cells. After quality assessment by Thermo NanoDrop 2000 and Agilent 2100 Bioanalyzer, RNA samples from both SMMC7721 STAT3 siRNA and SMMC7721 Scr siRNA cells were analyzed by GeneChip primeview human from GeneChem Co. (Shanghai, China). The analysis of data was run by PathArray^TM^.

### Cell apoptosis and viability detection

Cell apoptosis was determined by Capase3/7 activity assay with Caspase-Glo® 3/7 Assay System (Catalog No.: G8090, Promega, USA) according to the manufacturer's instructions. Cell viability was measured by MTT assays according to the manufacturer's recommendations.

### Cell migration and invasion assays

Cell migration and invasion assays were carried respectively with Corning ^®^ BioCoat^TM^ Control Cell Culture inserts and Biocoat Matrigel invasion chambers. HCC cells were dissociated with trypsin, washed with PBS, and then re-suspended as single-cell suspensions in DMEM medium with 0.1% FBS. Then HCC cells were seeded to the upper chamber with uncoated membrane or Matrigel-coated membrane to the bottom chamber with DMEM medium with 10% FBS. HCC cells on the upper level of the filter were cleaned out after 12 hours. The cells on the lower level of filter were fixed with 4% paraformaldehyde, dyed with crystal violet, and counted from 3 randomly selected fields under microscopy.

### Chromatin immunoprecipitation assay (ChIP)

HCC cells were cultured in 15cm plated to reach 80% confluence and then DNA was isolated. Bioruptor Pico sonicator was used to shear DNA. ChIP assay was conducted with EZ CHIP KIT (Catalog No.:17-371). DNA fragments precipitated were determined by PCR. The PCR primers to detect the promoter fragment of HIF-1α were Forward: TGAGTGAAGCAGTTCTCAGCAT, Reverse: GTACCTGTAGGTGCAGGGATT. The primers for the SNAI1 promoter fragment were Forward: GGCTCTGAGTGTTCTGTCCG, Reverse: GTGGCATTGACGAGGGAAAC.

### Luciferase reporter assay

MHCC97h cells were grown in 12-well plate and transfected respectively with pGL3‐based construct carrying different HIF-1α promoter fragments plus Renilla luciferase plasmid. Reporter activity was assessed by the luciferase assay kit (Promega, USA) and calculated as the ratio of luciferase intensity/renilla luciferase intensity.

### HCC xenograft experiments

The protocol of* in vivo* nude mouse experiments was approved by the Care and Use of the Animal Ethics Committee of the First Hospital of Xian Jiaotong University. A total of 20 male nude mice aged 3-4 weeks from Animal Experiment Center of Xian Jiaotong University were divided into 4 groups. 1×10^6^ Huh7 HIF-1α cells suspended in PBS implanted subcutaneously into the flank of each mouse as Huh7 HIF-1α group. As to Huh7 Vector group, 1×10^6^ Huh7 Vector cells were injected subcutaneously into the flank of each nude mouse. Doxorubicin (DOX) was injected intraperitoneally respectively every 2 days for 2 weeks to be DOX treatment group. And mice from placebo group was injected intraperitoneally with normal saline. After four weeks, all mice were sacrificed by cervical dislocation under anesthesia and HCC xenografts were harvested. Tumor sizes were assessed weekly using calipers and calculated with the following formula: volume = A × B^2^ × 0.52 (A, length; B, width; all measurements were in millimeters). The samples from HCC xenografts were assessed by IHC assay.

### Statistical analysis

All target protein expression levels in HCC tissues and matched adjacent liver tissues were analyzed by the Mann-Whitney U test. The correlation between p-STAT3 and HIF-1α, SNAI1, E-cadherin and Vimentin was studied with Spearman rank test. The Kaplan-Meier survival curves were compared using the log-rank test. The p-value of < 0.05 was significant. Multivariate analysis was conducted using SPSS Version 17.0 software (SPSS Inc., Chicago, IL, USA). PRISM 5 software (GraphPad, La Jolla, CA, USA) was used for all other statistical analyses.

## Results

### HCCs with increased expression of p-STAT3 suffered from the unfavorable post-TACE prognosis

**Figure 1:**
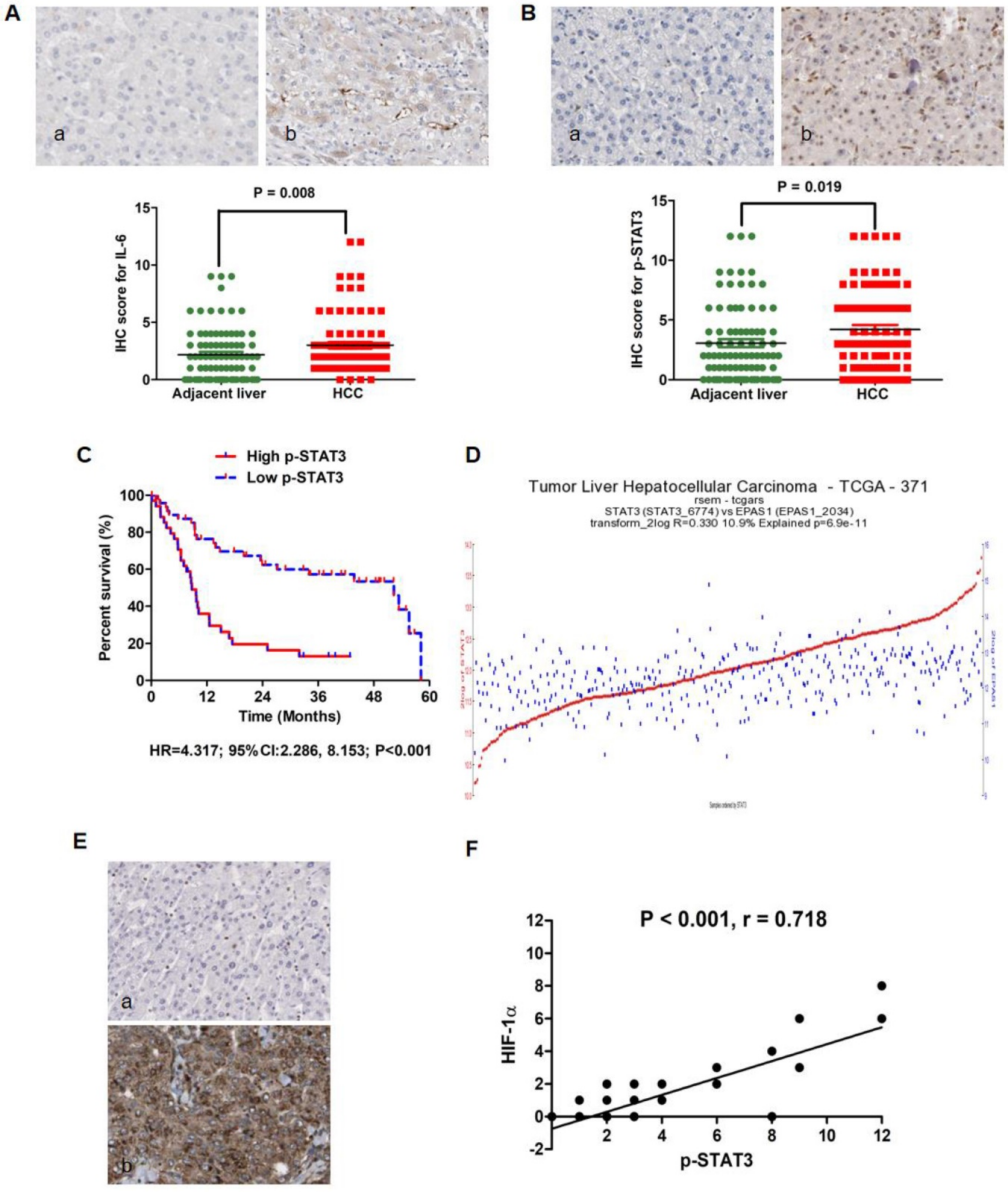
** IL-6/STAT3 pathway was hyperactivated in HCC tissues and related with up-regulation of HIF-1α positively. A.** IL-6 protein expression was found predominantly in the cytoplasm of HCC cells and positive in samples from 82 of 86 HCCs (95.3%) by IHC assay. Mann-Whitney U test confirmed that there was more IL-6 expression in HCC tissues than adjacent liver tissues. **B.** p-STAT3 protein was found located mainly in nucleus of tumor cells, while there was also detectable p-STAT3 protein in cytoplasm of tumor cells as assessed by IHC. Mann-Whitney U test also demonstrated that the expression of p-STAT3 in tumor samples was significantly higher than one in adjacent liver samples. **C.** By Log-rank test, comparison of survival curves revealed that HCCs from High p-STAT3 group suffered from remarkably worse prognosis after TACE than those from Low p-STAT3 group. **D.** The analysis of data from the Cancer Genome Atlas (TCGA) database displayed that there was apparently correlation between STAT3 mRNA and HIF-1α mRNA in 371 HCC samples (Fig.1D, r-value=0.393 p-value=3.6e-15 T-value=8.215). **E.** IHC staining assay exhibited that HIF-1α protein located in both cytoplasm and nucleus of tumor cells and Spearman rank correlation analysis that there was remarkably positive association between p-STAT3 and HIF-1α in HCC tissues.

Our previous investigation showed that IL-6/STAT3 signaling was aberrantly activated in HCC tissues and involved closely with the unfavorable outcome of HCCs after liver resection 9. Here, we detected the relationship between p-STAT3 expression in HCC tissues harvested by liver biopsy before TACE and post-TACE prognosis. As shown in Fig.1A, IL-6 protein expression was found predominantly in the cytoplasm of HCC cells and positive in samples from 82 of 86 HCCs (95.3%) by IHC staining assay. In 60/86 HCCs, IL-6 expression in tumor tissues was found stronger than one in adjacent liver tissues. And analysis of the IHC scores also revealed that there was more IL-6 expression in HCC tissues than adjacent liver tissues by Mann-Whitney U test (Fig.1A). The expression of p-STAT3 protein located mainly in nucleus of tumor cells, while there was also detectable p-STAT3 protein in cytoplasm of tumor cells (Fig.1B). Consistently, p-STAT3 expression was positive in tumor tissues from 67/86 HCCs (77.9%) and increased clearly in tumor tissues compared to adjacent liver tissues in 54/86 HCCs (62.8%). Mann-Whitney U test also demonstrated that the expression of p-STAT3 in tumor samples was significantly higher than one in adjacent liver samples (Fig.1B). Using the ratio of p-STAT3 expression in tumor/adjacent liver tissues as the cut-off value, HCCs with higher p-STAT3 expression in HCC tissues were classified into High p-STAT3 group and others were Low p-STAT3 group. As presented in Table 1, it was found that up-regulated p-STAT3 expression in HCC tissues was associated with HBV infection, larger tumor diameter, intrahepatic metastases, PVTT and tumor differentiation. By Log-rank test, comparison of survival curves revealed that HCCs from High p-STAT3 group suffered from remarkably worse prognosis after TACE than those from Low p-STAT3 group (Fig.1C, HR=4.317; 95%CI:2.286, 8.153; P < 0.001). The 1-year survival rate of High p-STAT3 group was 32.68% which was significantly lower than one of Low p-STAT3 group (76.36%). The median survival time of patients from High p-STAT3 group was 8.614 months, whereas median survival time of Low p-STAT3 group was 52.36 months. These data indicated that hyperactivation of IL-6/STAT3 pathway in tumor tissues predicated poor outcome of HCC patients after TACE. As shown in Table 2, univariate analysis of overall survival time showed that high p-STAT3 expression in tumor tissue, advanced TNM staging, intrahepatic metastases, PVTT and TACE times were the poor prognostic factors. Furthermore, multivariate Cox proportional hazards regression test identified high p-STAT3 expression in tumor tissue, advanced TNM staging, PVTT and TACE times as the independent predictive factors.

HIF-1α has been verified aberrantly up-regulated in HCC tissues after TACE by several studies^39^-^41^, and exerted the angiogenic effect through mediating pro-angiogenic factors including VEGF, PDGF and PLGF, which ultimately promoted the growth and metastases of cancer cells after TACE^42^. The Cancer Genome Atlas (TCGA) database showed that there was apparently correlation between STAT3 mRNA and HIF-1α mRNA in 371 HCC samples (Fig.1D, r-value=0.393 p-value=3.6e-15 T-value=8.215). Based on this finding, we examined the correlation between p-STAT3 and HIF-1α protein expression in tumor samples. IHC staining assay exhibited that HIF-1α protein located in both cytoplasm and nucleus of tumor cells (Fig.1E). Spearman rank correlation analysis that there was remarkably positive association between p-STAT3 and HIF-1α in HCC tissues. After searching the TCGA database, although we did not found significant difference of HIF-1α mRNA expression in between 374 HCC and 50 normal liver tissues (Fold-change = 1.48, P = 0.16, Supplementary Fig 1A), HCC patients with higher HIF-1α mRNA expression in tumor tissues suffered from the worse prognosis than those with lower HIF-1α in tumor tissues compared to adjacent liver tissues (HR = 1.73, P = 0.003, Supplementary Fig 1B). These suggested also that HIF-1α was involved closely in HCC progression. Furthermore, TCGA database showed that there was no significant difference found about STAT3 mRNA expression in between 374 HCC and 50 normal liver tissues (Fold-change = 0.9, P = 0.061, Supplementary Fig 2A). Comparison of survival curves displayed that no apparent difference was found between Low STAT3 patients who had less STAT3 mRNA expression in HCC tissues and High STAT3 patients with more STAT3 mRNA in HCC tissues compared to adjacent liver tissues (HR = 0.98, P = 0.93, Supplementary Fig 2B). It indicated that p-STAT3, rather than STAT3, could play an important role in initiation and progression of HCC.

### IL-6/STAT3 pathway was required for hypoxia-induced aberrant activation of HIF-1 signaling in HCC cells

**Figure 2:**
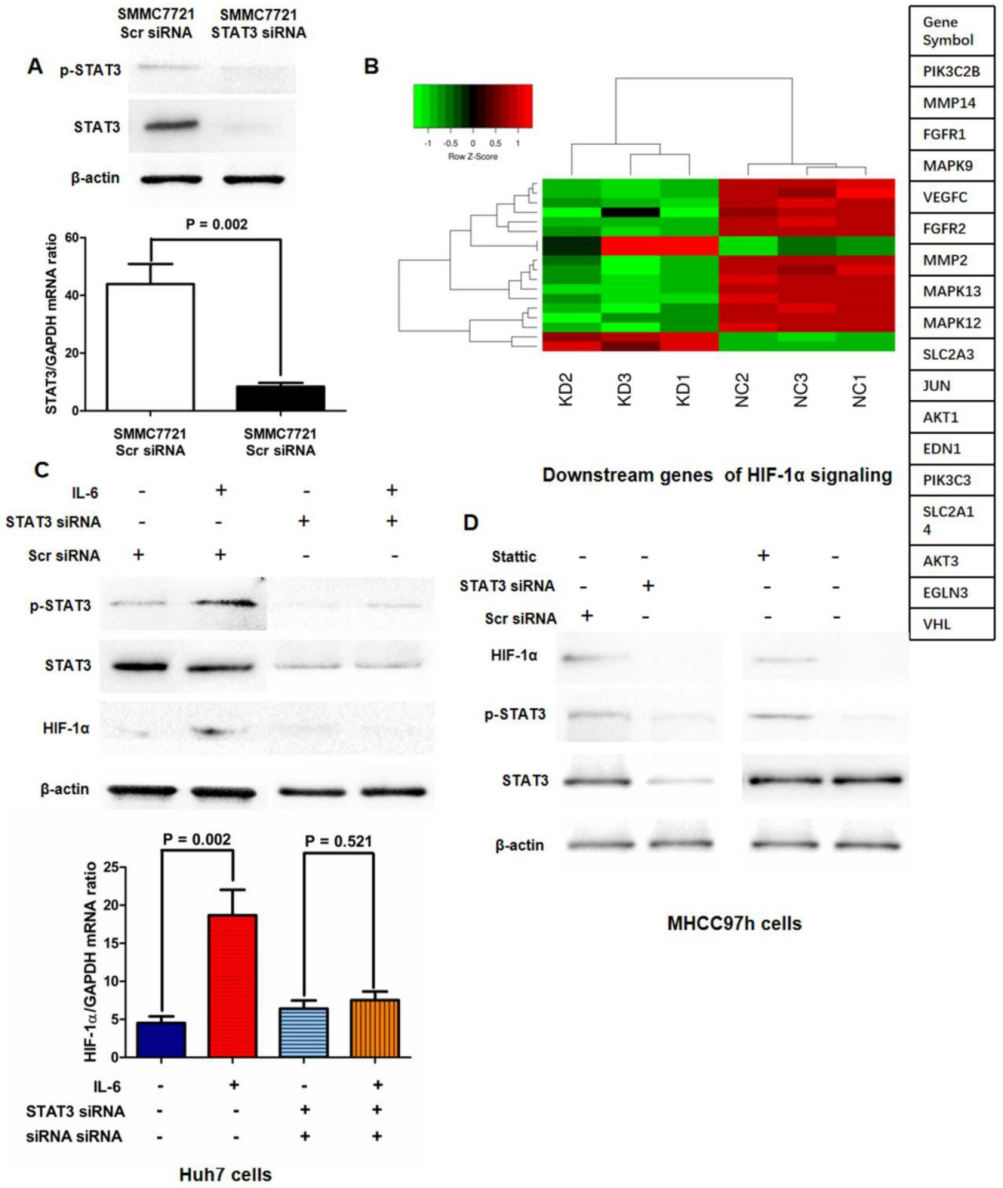
** Activation of IL-6/STAT3 pathway played an important role in hypoxia-induced HIF-1α up-regulation. A.** HIF-1α expression was verified to be down-regulated in SMMC7721 STAT3 siRNA cells compared to SMMC7721 control siRNA cells at the levels of mRNA and protein by both qRT-PCR and Western immunoblotting. **B.** mRNA expression microarray assay revealed that knockdown of STAT3 in SMMC7721 cells leaded to the down-regulation of HIF-1α signaling. **C.** Both qRT-PCR and Western immunoblotting assays displayed that IL-6 treatment increased expression of HIF-1α in Huh7 cells with up-regulation of p-STAT3 significantly, and after silencing STAT3, IL-6 did not reinforce HIF-1α expression in Huh7 cells any more. **D.** Both knockdown of STAT3 and the treatment of p-STAT3 inhibitor Stattic resulted in down-regulation of HIF-1α expression in MHCC97h cells.

Based on the relationship between p-STAT3 and HIF-1α found in clinical samples, we sought to figure out whether IL-6/STAT3 pathway mediated HIF-1α expression in HCC cells. We abolished the STAT3 expression by targeted siRNA sequences and run the mRNA expression Chip assay. As shown in Fig.2A, HIF-1α expression was verified to be down-regulated in SMMC7721 STAT3 siRNA cells compared to SMMC7721 control siRNA cells at the levels of mRNA and protein. Moreover, mRNA expression microarray assay revealed that knockdown of STAT3 in SMMC7721 cells leaded to the down-regulation of HIF-1α signaling (Fig.2B). The further qRT-PCR and Western immunoblotting assays verified that IL-6 treatment increased expression of HIF-1α in Huh7 cells with up-regulation of p-STAT3 (Fig.2C). Both knockdown of STAT3 and the treatment of p-STAT3 inhibitor Stattic resulted in down-regulation of HIF-1α expression in MHCC97h cells (Fig.2D). And IL-6 treatment did not enhance HIF-1α expression in the STAT3-deficient Huh7 cells significantly (Fig.2C). It indicated that IL-6/STAT3 pathway mediated positively HIF-1α in HCC.

**Figure 3:**
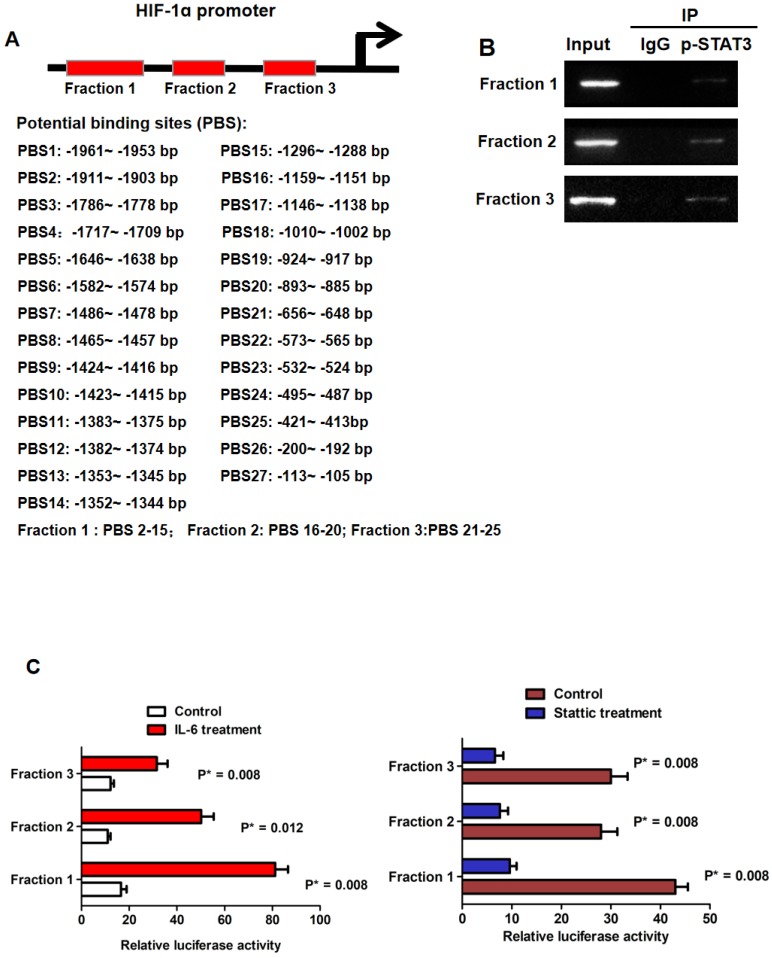
** IL-6/STAT3 pathway increased HIF-1α expression directly via p-STAT3 binding with HIF-1α promoter. A.** Bio-informatic analysis revealed that there were 27 potential p-STAT3 DNA binding sites found in the promoter of HIF-1α. **B.** Based on the location of these 27 potential binding sites, we divided the HIF-1α promoter into 3 regions which could be bound by p-STAT3 protein including Fraction 1, Fraction 2 and Fraction 3. ChIP assay confirmed that p-STAT3 protein was bound with all three fractions respectively. **C.** Luciferase reporter assessment displayed that IL-6 treatment enhanced the occupancy of p-STAT3 protein on all three fractions of HIF-1α promoter in Huh7 cells. And the p-STAT3 inhibitor Stattic repressed the bindings between p-STAT3 protein and these 3 fragments in MHCC97h cells.

In silico analysis revealed that there were 27 potential p-STAT3 DNA binding sites found in the promoter of HIF-1α as shown in Fig.3A. Based on the location of these 27 potential binding sites, we divided the HIF-1α promoter into 3 regions which could be bound by p-STAT3 protein including Fraction 1, Fraction 2 and Fraction 3. With the help of primes against these 3 fragments respectively, we conducted ChIP assay and found all three fractions were detected in chromatin fragments pulled down by the anti-p-STAT3 antibody (Fig.3B). To figure out whether the binding of p-STAT3 protein activated HIF-1α promoter, we built the 3 kinds of HIF-1α luciferase promoter vector constructs including Fraction 1, 2 and 3 respectively. IL-6 treatment reinforced the luciferase activity of all 3 reporters remarkably in Huh7 cells and the p-STAT3 inhibitor Stattic repressed the activity of these 3 reporters in MHCC97h cells (Fig.3C). The data here suggested that phosphorylation of STAT3 induced by IL-6 enhanced HIF-1α expression in HCC cells via binding with the promoter.

**Figure 4:**
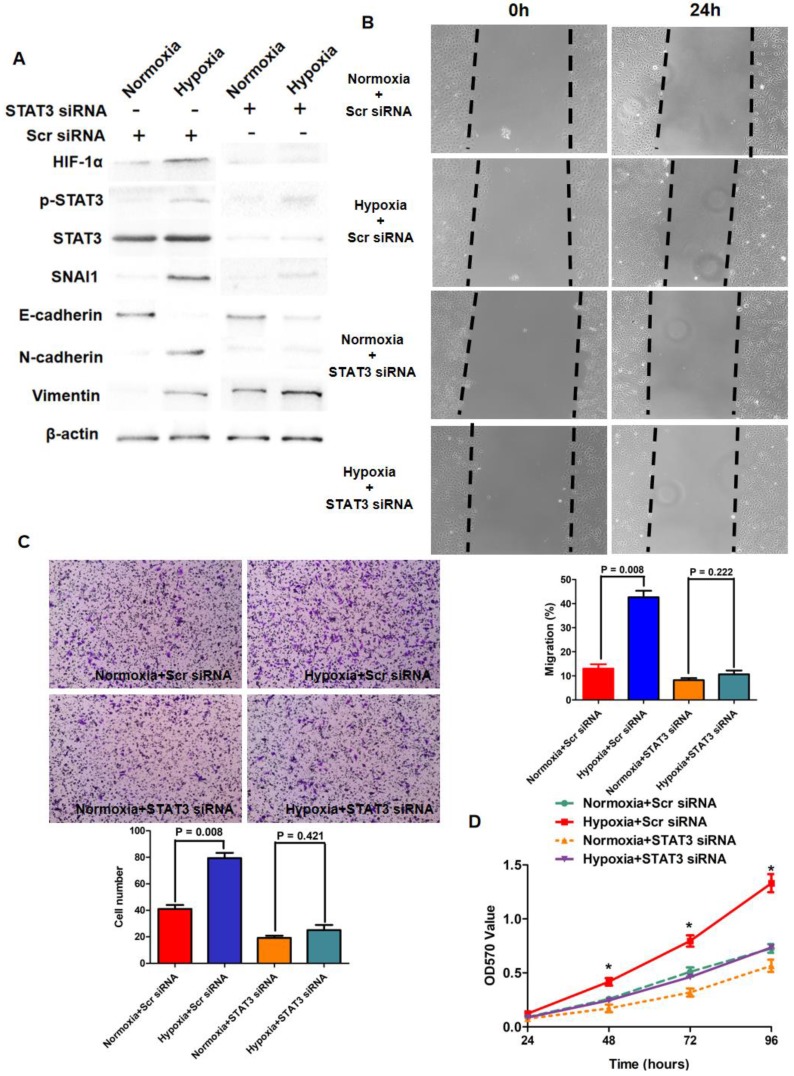
** IL-6/STAT3 pathway was involved closely in hypoxia-driven EMT phenotype of HCC cells. A.** Hypoxia culture increased the expression of HIF-1α, SNAI1, N-cadherin, Vimentin, repressed E-cadherin expression, and enhanced the phosphorylation of STAT3 protein in Huh7 cells. Knockdown of STAT3 by siRNA sequences decreased p-STAT3 expression and inhibited the impact of hypoxia culture on the expression of HIF-1α and EMT markers. **B.** Wound healing assay showed that hypoxia culture reinforced the migratory capacity of Huh7 cells, and after silencing STAT3, hypoxia culture did not increase migration of Huh7 cells. **C.** As assessed by Transwell chamber coated with Matrigel, hypoxia culture promoted invasion of Huh7 cells apparently. And knockdown of STAT3 suppressed the pro-invasion effect of hypoxia in Huh7 cells. **D.** MTT assay showed that hypoxia enhanced the viability of Huh7 cells and silencing STAT3 attenuated the pro-growth action of hypoxia in Huh7 cells.

It has been considered typically that Hypoxia resulted in lack of sufficient oxygen which impaired the hydroxylation and proteasomal degradation of HIF-1α protein. Because IL-6/STAT3 signaling was hyper-activated in HCC tissues secondary to TACE treatment and p-STAT3 reinforced HIF-1α expression in HCC cells, we next sought to figure out whether IL-6/STAT3 pathway played the critical role in up-regulation of HIF-1α driven by hypoxia. As shown in Fig.4A, hypoxia culture (1% O_2_) increased HIF-1α expression significantly in Huh7 cells compared to normoxia culture (21%). Furthermore, silencing STAT3 by siRNA sequences inhibited the pro-regulatory effect of hypoxia (Fig.4A). These indicated that p-STAT3 was critical for increased HIF-1α expression induced by hypoxia in the manner of modifying transcription.

### p-STAT3 was involved closely in hypoxia-induced EMT phenotype of HCC cells via HIF-1α /SNAI1 axis

Growing body of evidences revealed that EMT contributed to hypoxia promoting growing and metastases of cancer cells^43^-^46^, however, the underlying mechanism has not been uncovered completely. Hence, we determined initially whether inflammation secondary to TACE treatment was attributed to EMT-like alteration of HCC cells via STAT3/HIF-1α/SNAI1 axis in hypoxia microenvironment. Hypoxia culture for Huh7 cells down-regulated epithelial marker E-cadherin and up-regulated mesenchymal marker N-cadherin and Vimentin while increased the expression of both HIF-1α and SNAI1, as assessed as Western immunoblotting assay (Fig.4A). It was interested to observe that hypoxia also reinforced the phosphorylation of STAT3 (Fig.4A). The migration and invasion capacities of Huh7 were promoted clearly (Fig.4B and 4C). The viability of Huh7 cells was also found enhanced by hypoxia culture (Fig.4D). However, after knockdown of STAT3 with siRNA sequences, no prominent EMT-like alternation was detected in Huh7 cells and HIF-1α expression was not increased notably (Fig.4A). Additionally, hypoxia did not regulate the migration, invasion and viability of STAT3-deficient Huh7 cells clearly (Fig.4B, 4C and 4D). It suggested that phosphorylation of STAT3 was critical for HIF-1α-induced EMT of HCC cells under hypoxia condition.

**Figure 5:**
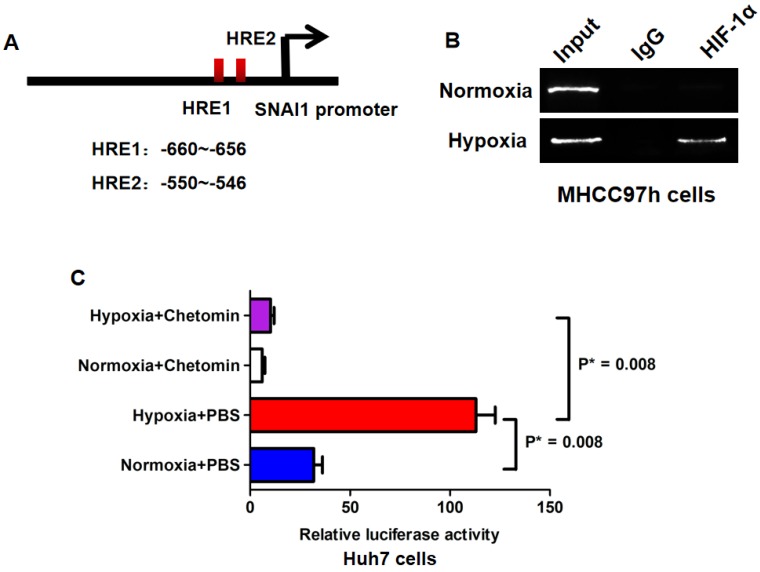
** HIF-1α enhanced SNAI1 expression via directly binding to its promoter**. **A.** There were two hypoxia response elements (HREs) found in the promoter of SNAI1 after searching UCSC Genome database. **B.** ChIP assay revealed that HIF-1α protein was bound with the -691~ -405 bp fragment of SNAI1 promoter in MHCC97h cells and hypoxia culture strengthened the occupancy of HIF-1α in the SNAI1 promoter. **C.** In luciferase reporter assay, it was found that hypoxia culture increased significantly the luciferase activity of reporter in Huh7 cells, however, the treatment of HIF-1α inhibitor, Chetomin suppressed the luciferase activity in Huh7 cells, even in hypoxia condition.

Bioinformatics analysis revealed that there were two hypoxia response elements (HREs) in the promoter of SNAI1 (Fig.5A). ChIP assay confirmed that HIF-1α protein was bound with the -691~ -405 bp fragment of SNAI1 promoter in MHCC97h cells (Fig.5B) and hypoxia culture augmented the occupancy of HIF-1α in the SNAI1 promoter. The -691~ -405 bp fragment was reconstituted into the pGL3-basic luciferase reporter vector and the reporter vector was transfected into Huh7 cells. As shown in Fig.5C, hypoxia culture increased dramatically the luciferase activity of reporter in Huh7 cells, however, the treatment of HIF-1α inhibitor, Chetomin, at the concentration of 150nM, suppressed the luciferase activity in Huh7 cells, even in hypoxia condition.

### Suppression of HIF-1α pathway sensitized the anti-HCC effect of doxorubicin *in vitro* and* in vivo*

**Figure 6:**
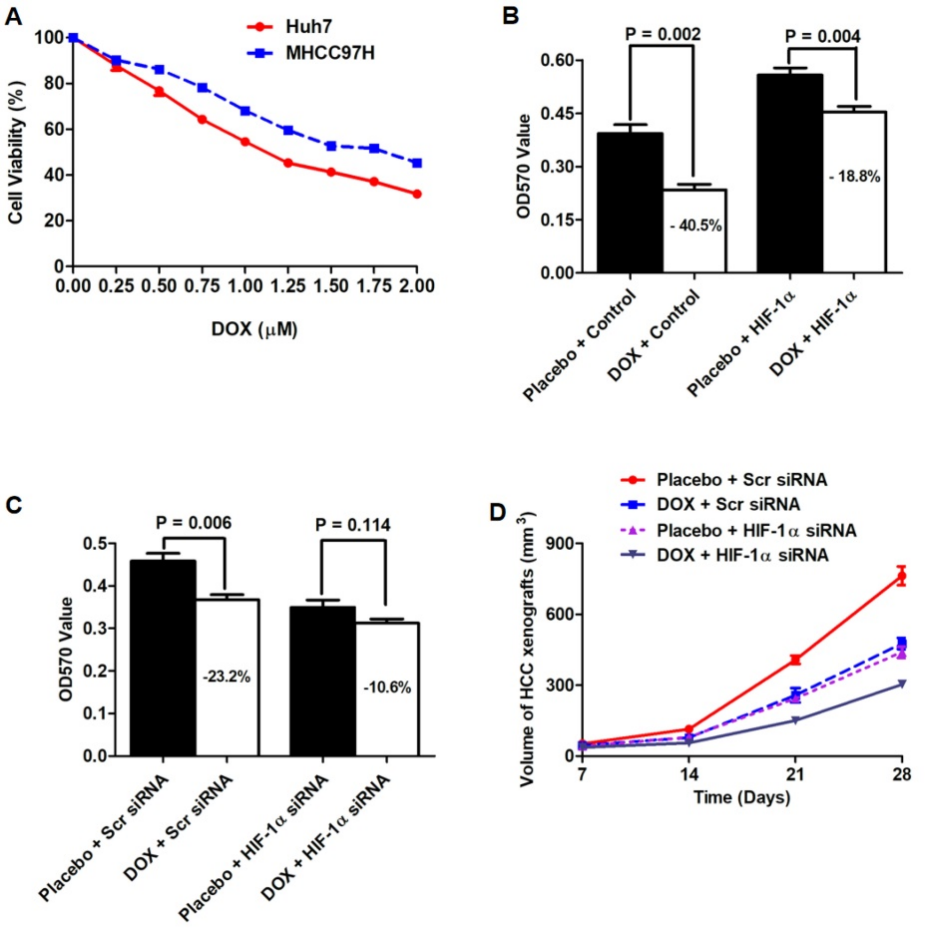
** Knockdown of HIF-1α sensitized the anti-HCC effect of DOX *in vitro* and *in vivo*. A.** MTT assay showed that HCC cells were repressed in a dose-dependent manner. **B.** MTT assay revealed that DOX administration (0.75 μM) leaded to a significant drop in cell viability in Huh7 cells, which was suppressed by HIF-1α up-regulation. **C.** The cytotoxicity of DOX on MHCC97h cells was also enhanced by siRNA-induced depletion of endogenous HIF-1α. **D.** HCC xenografts driven from Huh7 HIF-1α cells (PBS group) were dramatically larger than those from Huh7 Vector cells (PBS group). After DOX administration, the size of HCC xenografts from Huh7 HIF-1α cells were larger than those from Huh7 Vector cells.

Doxorubicin (DOX), which was an anthracycline based agent, was able to trigger HCC cell apoptosis via inducing DNA damage and has been widely used in TACE treatment for HCC. However, the emergency of DOX resistance blocked the successful TACE treatment of HCC^47^. Here, we sought to figure out whether HIF-1α up-regulation impacted the cytotoxicity of DOX to HCC cells. MTT assay was performed to measure DOX-cytotoxicity after both Huh7 and MHCC97h cells were treated with DOX at 9 kinds of different concentrations including 0, 0.25, 0.50, 0.75, 1.00, 1.25, 1.50, 1.75 and 2.00 μM. As presented in Fig.6A, both HCC cells were repressed in a dose-dependent manner. The results of MTT assay between control group (0 μM DOX) and other concentrations of DOX (range: 0.25-2.00 μM) were compared by Mann-Whitney test and the value of P < 0.05 was considered as statistically significant. As shown in Fig.6B, MTT assay revealed that DOX administration (0.75 μM) leaded to a significant drop in cell viability in Huh7 cells, which was suppressed by HIF-1α up-regulation. As assessed by MTT, the cytotoxicity of DOX on MHCC97h cells was also enhanced by siRNA-induced depletion of endogenous HIF-1α (Fig.6C).

Huh7 cell nude mouse xenograft was established to determine whether HIF-1α attenuated the anti-HCC effect of DOX. A total of twelve nude mice were injected with Huh7 HIF-1α cells as HIF-1α group, while another 12 nude mice were injected with Huh7 Vector cells to be control group. In HIF-1α group, 6 mice were injected with DOX at the concentration of 0.75 μM intraperitoneally (DOX group), whereas another 6 mice were treated with PBS buffer (PBS group). Similarly, mice from control group were also divided into two groups, DOX group (injection with DOX at 1 mg/kg per mouse each time intraperitoneally) and PBS group (injection with PBS intraperitoneally). As shown in Fig.6D, without DOX treatment, HCC xenografts driven from Huh7 HIF-1α cells (PBS group) were dramatically larger than those from Huh7 Vector cells (PBS group), which indicated that HIF-1α could reinforce the growth of HCC cells *in vivo*. Furthermore, after DOX administration, the size of HCC xenografts from Huh7 HIF-1α cells were larger than those from Huh7 Vector cells, which verified that HIF-1α repressed the anti-HCC of DOX *in vivo*.

## Discussion

HCC was usually found at the intermediate/advanced stage—closely related with the development of cirrhosis decompensation, at which the available therapy options were limited and ineffective. Diverse potentially therapeutic approaches have been determined in HCC patients with intermediate stage HCC, though several treatment options showed some efficacy, TACE is the only treatment with a consistent survival benefit for those HCC patients 5, 48 and has been currently widely accepted in clinical guidelines. TACE exerts the anti-HCC effect based on two prominent mechanisms: the cytotoxic chemotherapeutic effect and the ischemic effect driven from angiographic selective arterial occlusion of tumor blood supply. The advantage of trans-arterial chemotherapy is to achieve the highest local concentration of the chemotherapy drugs in HCC lesion with the help of Lipiodol or drug-eluting embolization microspheres (beads) for a longer period of time. Unfortunately, because that blood supply of HCC is predominantly from hepatic artery (75%-95%), portal vein provides tumor lesions with blood partially, embolization of hepatic artery did not bring about complete ischemic necrosis of HCC lesion and consequently brought about postembolization hypoxia which has been considered to be attributed to local chemotherapy failure during TACE. However, the underlying mechanisms remain unclear. Tumor hypoxia has been verified to be correlated with genetic instability, disease progression and metastasis, and inhibition of cancer response to cytotoxic and targeted therapies^49^. HIF family is central in the cellular responses of both tumor and stromal compartments in cancer microenvironment to hypoxia^50^. HIF-1α expression in serum from HCC patients was magnificently increased 1 day after receiving TACE and the peak values were observed at the 7th day after TACE, which was followed by a subsequent decrease with sustained higher level than pre-TACE^25^. It seemed that HIF-1α could play an important role in hypoxia-induced malignant biological behavior of HCC cells after TACE. Currently, the precise mechanisms that TACE causes up-regulation of HIF-1αand HIF-1α promotes metastatic capacity and represses effect of cytotoxic drug in HCC are still vague.

The IL‑6/STAT3 pathway has a key role in hepatocarcinogenesis and HCC progression via mediating a variety of cell signalings 9, 47. Previous study found that liver acute inflammatory response increased IL-6 expression in liver tissues^51^. And TACE resulted in significant acute inflammatory response in both HCC and adjacent liver tissues, hence, we raised a hypothesis that TACE-driven activation of IL-6/STAT3 pathway modulated HIF-1α-induced EMT, and consequently accelerated the growth and metastases of residual HCC cells and attenuated the anti-tumor effect of chemotherapy drugs, which was attributed to TACE treatment failure. In this investigation, analysis of the relationship between p-STAT3 expression in HCC tissues before TACE and post-TACE survival by comparison of Kaplan-Meier curves showed that HCCs with hyperactivation of IL-6/STAT3 signaling suffered from the remarkably worse prognosis after TACE. Furthermore, IHC assay here revealed the marked positive association between p-STAT3 and HIF-1α, which was validated by analysis of the data from TCGA database. Next, we determined whether IL-6/STAT3 signaling was involved in up-regulated HIF-1α expression by hypoxia. In the* in vitro* experiments, knockdown of STAT3 was found to decrease HIF-1α expression in HCC cells by both Western immunoblotting and mRNA microarray. IL-6 treatment did not increased HIF-1α expression anymore after repression of STAT3 with the specific inhibitor or siRNA. And the bio-informatic analysis revealed that there were more than 20 potential p-STAT3 DNA-binding sites in the HIF-1α promoter, and then both ChIP and luciferase reporter assays confirmed that p-STAT3 could be bound with the promoter of HIF-1α gene and increase HIF-1α expression. Interestingly, interruption of IL-6/STAT3 pathway by siRNAs sequences against STAT3 reversed the pro-regulatory effect of hypoxia on HIF-1α expression in HCC cells dramatically.

In this study, hypoxia was verified again to induce significant EMT phenotype of HCC cells, while increased the expression of p-STAT3, HIF-1α and SNAI1. Then, we silenced the expression of STAT3 and found that hypoxia did not induce EMT phenotype of HCC cells and increase both HIF-1α and SNAI1, which indicated that p-STAT3 played a critical role in HIF-1α-driven EMT under hypoxia. The similar tendency of HIF-1α and SNAI1 found in both hypoxia experiments and STAT3 knockdown experiments raised the question whether SNAI1 was controlled by HIF-1α and contributed into EMT induced by HIF-1α. In silico analysis revealed that several HRE located in the SNAI1 promoter and ChIP assay confirmed that HIF-1α could increase directly SNAI1 expression via binding with SNAI1 promoter. Luciferase reporter assessments showed that hypoxia reinforced the combination between HIF-1 protein and SNAI1 promoter, which suggested strongly that HIF-1α was critical to SNAI1 up-regulation induced by hypoxia. Finally, by both the *in vitro* and *in vivo* experiments, we found in this study that IL-6/STAT3 pathway activation attenuated the anti-HCC effect of DOX dramatically which was used most commonly in TACE. It indicated that IL-6/STAT3 pathway activated aberrantly by hypoxia and inflammation secondary to TACE treatment was attributed to local chemotherapy failure of TACE.

**Figure 7:**
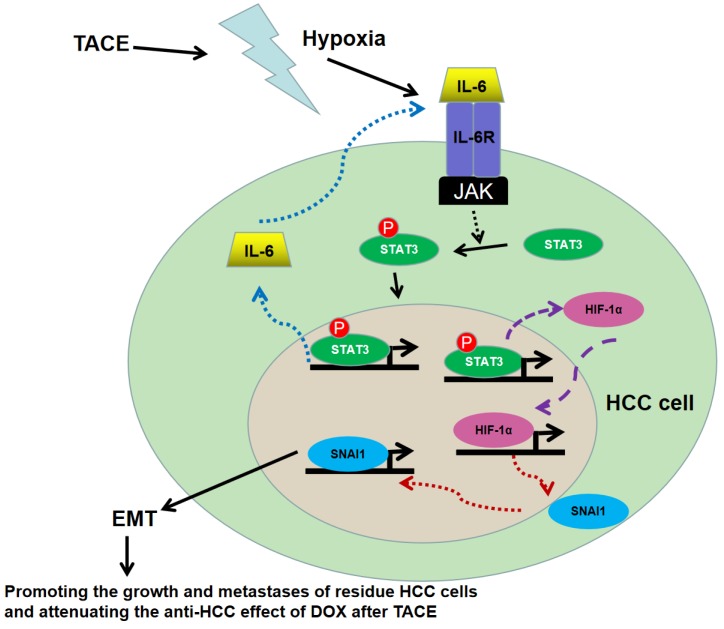
** Working model of the role of IL-6/STAT3 pathway on hypoxia promoting HCC progression after TACE.** Hypoxia secondary to TACE hyperactivated IL-6/STAT3 signaling on residue HCC cells. More p-STAT3 was trans-located into nucleus of HCC cells and bound with the promoter of IL-6 and HIF-1α respectively, which kept the sustained activation of IL-6/STAT3 signaling and increased the expression of HIF-1α. Consequently, HIF-1α up-regulated SNAI1 expression, induced EMT, and then accelerated the growth and metastases of the residue HCC cells and attenuated the anti-HCC effect of DOX during TACE.

In summary, this study found that HCC patients with hyperactivation of IL-6/STAT3 pathway suffered from the unfavorable post-TACE prognosis. The mechanism studies here revealed that hypoxia secondary to TACE could activate IL-6/STAT3 pathway which was able to increase HIF-1α expression directly acting as a transcription factor, consequently up-regulated SNAI1 and finally induced EMT, as shown in Fig.7. The EMT phenotype driven by IL-6/STAT4/HIF-1α/SNAI1 axis promoted the growth and metastases of residue HCC cells and attenuated the anti-HCC effect of DOX, which resulted in the failure of TACE treatment. Because inflammation has been considered as the key coactivator of IL-6/STAT3 signaling, it suggested that inflammation secondary to TACE was another critical factor to HIF-1α induced EMT and HCC relapse and metastases besides hypoxia. And the anti-inflammation treatment could be an option for post-TACE treatment to relieve side effect and inhibit tumor progression.

## Supplementary Material

Supplementary figures and tables.

## Figures and Tables

**Table 1:** Clinical characteristics and prognostic factors analysis for 86 HCCs

Variables		No. of patients		χ2	P value
		High p-STAT3 group	Low p-STAT3 group		
**Age (years)**	≤50	15	8	0.167	0.683
	>50	44	19		
**Gender**	Female	20	13	1.591	0.207
	Male	39	14		
**Liver cirrhosis**	No	7	2	0.393	0.531
	Yes	52	25		
**AFP level (ng/mL)**	≤400	12	9	1.695	0.193
	>400	47	18		
**HBV infection**	No	18	15	4.914	0.027
	Yes	41	12		
**TNM**	I+II	9	4	0.003	0.958
	III+IV	50	23		
**Tumor diameter (cm)**	≤5	17	15	5.670	0.017
	>5	42	12		
**Intrahepatic metastases**	Single	13	12	4.512	0.034
	Multiple	46	15		
**PVTT**	Present	43	12	6.498	0.011
	Absent	16	15		
**Tumor differentiation**	I+II	19	15	4.216	0.039
	III+IV	40	12		
**TACE times**	1	24	9	0.423	0.516
	>1	35	18		

**Table 2:** Cox proportional-hazard regression analysis of the relationship between clinicopathologic parameters and overall survival rate of 86 HCCs after TACE

Clinicopathologic Parameters	Unvariate Analysis			Multivariate Analysis	
	RR (95% CI)	P-value		RR (95% CI)	P-value
High p-STAT3 expression in tumor tissue	4.317(2.286- 8.153)	< 0.001		2.292(1.259-4.365)	< 0.001
Advanced TNM staging	3.013(1.689-4.425)	0.008		2.652(1.328-3.328)	0.007
Intrahepatic metastases	1.377(1.082-2.185)	0.032		0.925(0.329-2.022)	0.062
PVTT	2.357(1.587-3.357)	0.005		2.198(1.368-4.258)	0.017
TACE times	3.025(1.787-5.325)	< 0.001		2.558(1.275-5.658)	0.022

## Abbreviations

IL-6: Interleukin 6
HCC: Hepatocellular carcinoma
IHC: Immunohistochemistry
STAT3: Signal transducer and activator of transcription 3
HIF-1α: Hypoxia-inducible factor 1-alpha
TNM: Tumor-node- metastasis
TACE: Transarterial chemoembolization
DOX: Doxorubicin
EMT: Epithelial-mesenchymal transition
qRT-PCR: Quantitative reverse transcription polymerase chain reaction
PBS: Phosphate Buffered Saline
FBS: Fetal bovine serum
iTRAQ: Sobaric tags for relative and absolute quantitation
RFS: Recurrence-free survival
OS: Overall survival
mRECIST: Modified response evaluation criteria in solid tumors
