# Supplementary material for: Hyperactivation of IL-6/STAT3 pathway leaded to the poor prognosis of post-TACE HCCs by HIF-1α/SNAI1 axis-induced epithelial to mesenchymal transition
Source: J Cancer. 2020 Jan 1;11(3):570–82. doi: 10.7150/jca.35631 (PMC6959052; doi:10.7150/jca.35631)
Supplement: Supplementary file 1 — Supplementary figures and tables. [file jcav11p0570s1.pdf]

Supplementary Fig.1

Supplementary Fig.1 Analysis from the TCGA database showed that HCC patients with higher HIF-1α expression in HCC compared to adjacent liver tissues suffered from significantly worse prognosis than those with lower HIF-1α (B), though there was no difference of HIF-1α between HCC and normal liver tissues (A).

A

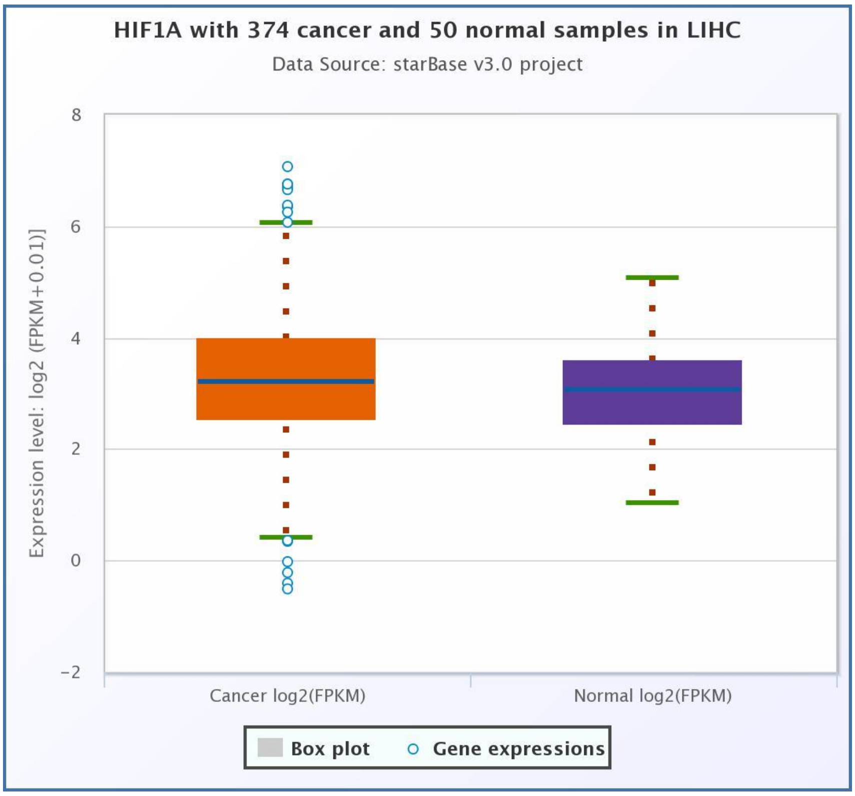

B

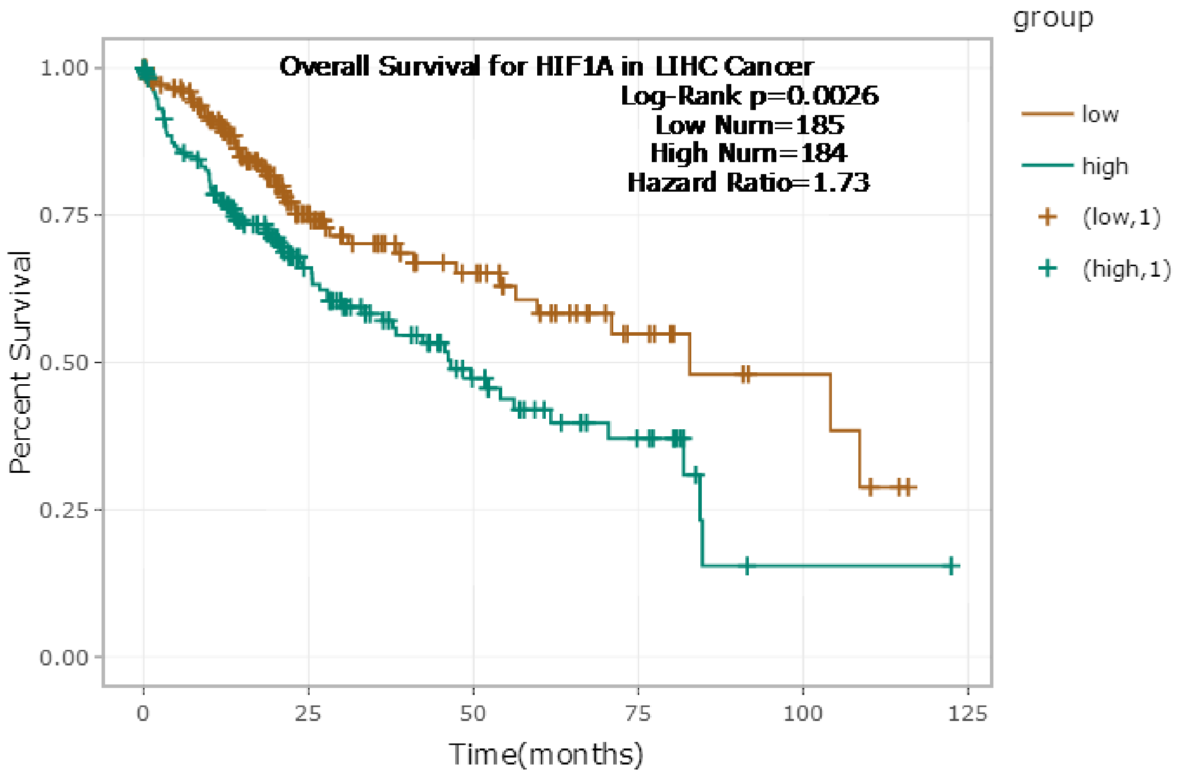
Overall Survival for HIF1A in LIHC Cancer
Log-Rank p=0.0026
Low Num=185
High Num=184
Hazard Ratio=1.73
Percent Survival
1.00
0.75
0.50
0.25
0.00
0
25
50
75
100
125
Time(months)
group
low
high
(low,1)
(high,1)

Supplementary Fig.2

Supplementary Fig.2 Analysis from the TCGA database displayed that there was no difference of STAT3 mRNA between HCC and normal liver tissues (A), and no apparent difference was found between Low STAT3 patients who had less STAT3 mRNA expression in HCC tissues and High STAT3 patients with more STAT3 mRNA in HCC tissues compared to adjacent liver tissues (B).

A

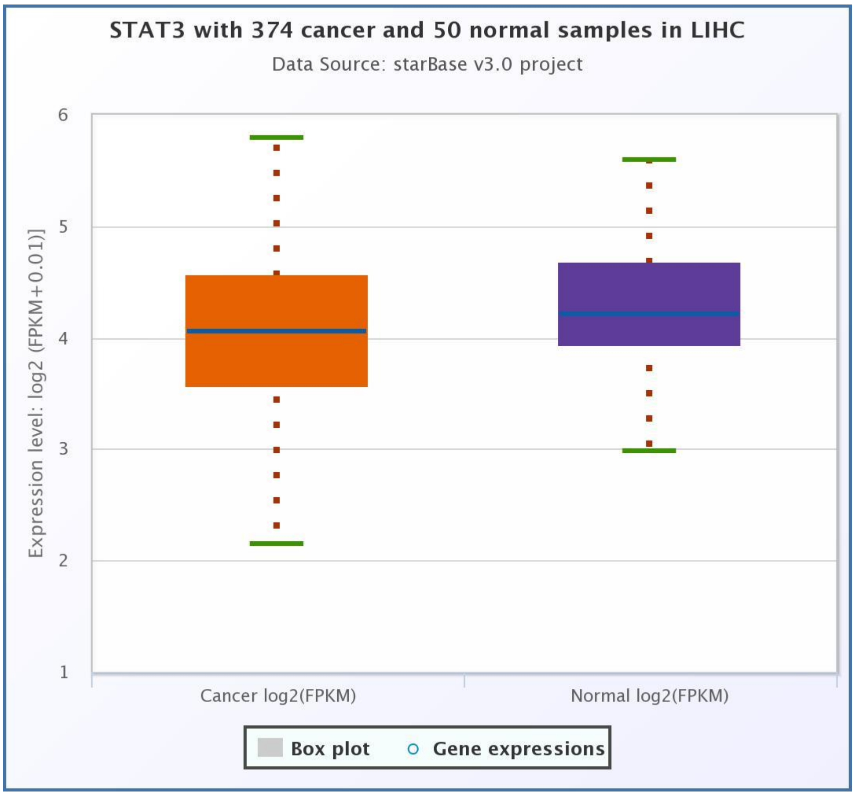

B

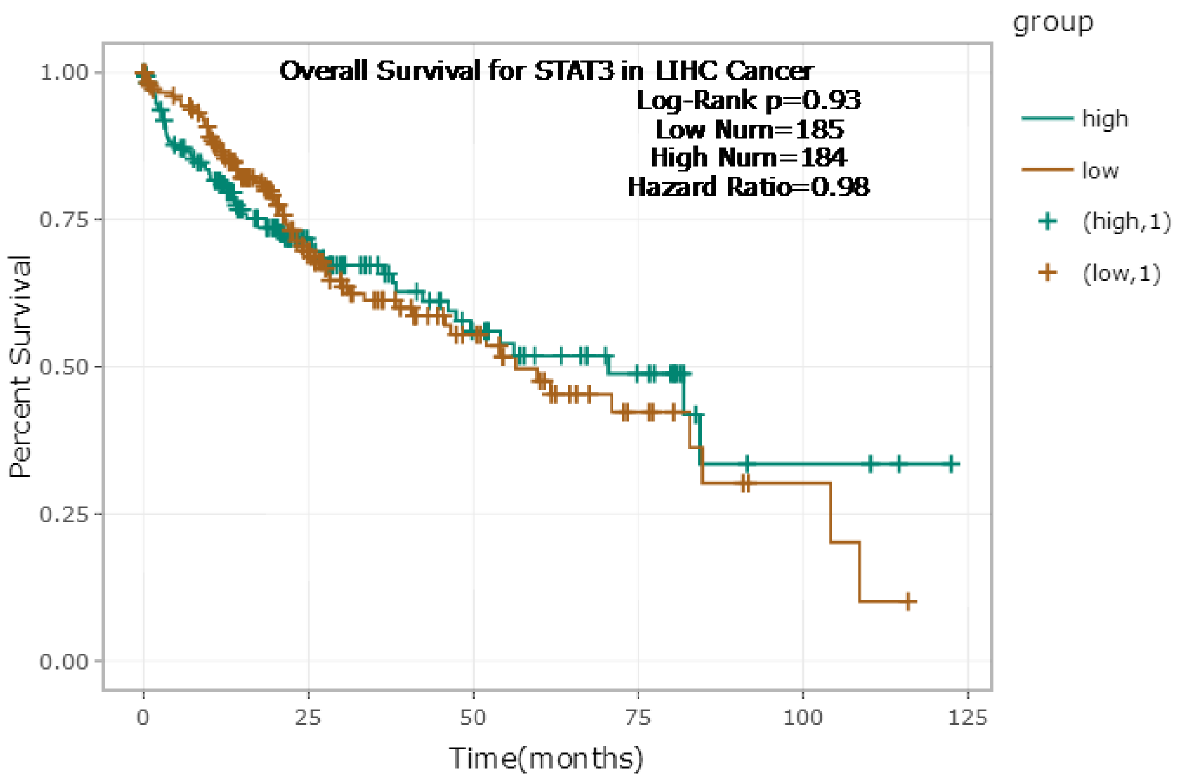
Overall Survival for STAT3 in LIHC Cancer
Log-Rank p=0.93
Low Num=185
High Num=184
Hazard Ratio=0.98
group
high
low
(high,1)
(low,1)
Percent Survival
1.00
0.75
0.50
0.25
0.00
0
25
50
75
100
125
Time(months)
